# Therapeutic prospects of nectin-4 in cancer: applications and value

**DOI:** 10.3389/fonc.2024.1354543

**Published:** 2024-03-28

**Authors:** Kaiyue Li, Yujing Zhou, Maolin Zang, Xin Jin, Xin Li

**Affiliations:** ^1^Department of Nuclear Medicine, Qilu Hospital of Shandong University, Jinan, Shandong, China; ^2^Department of Urology, Qilu Hospital of Shandong University, Jinan, Shandong, China; ^3^Imaging Center, Jinan Third People’s Hospital, Jinan, Shandong, China

**Keywords:** cancer, Nectin-4, therapy, biomarker, ADC

## Abstract

Nectin-4 is a Ca^2+^-independent immunoglobulin-like protein that exhibits significantly elevated expression in malignant tumors while maintaining extremely low levels in healthy adult tissues. In recent years, overexpression of Nectin-4 has been implicated in tumor occurrence and development of various cancers, including breast cancer, urothelial cancer, and lung cancer. In 2019, the Food and Drug Administration approved enfortumab vedotin, the first antibody–drug conjugate targeting Nectin-4, for the treatment of urothelial carcinoma. This has emphasized the value of Nectin-4 in tumor targeted therapy and promoted the implementation of more clinical trials of enfortumab vedotin. In addition, many new drugs targeting Nectin-4 for the treatment of malignant tumors have entered clinical trials, with the aim of exploring potential new indications. However, the exact mechanisms by which Nectin-4 affects tumorigenesis and progression are still unclear, and the emergence of drug resistance and treatment-related adverse reactions poses challenges. This article reviews the diagnostic potential, prognostic significance, and molecular role of Nectin-4 in tumors, with a focus on clinical trials in the field of Nectin-4-related tumor treatment and the development of new drugs targeting Nectin-4.

## 1 Introduction

Tumor-specific biomarkers encompass a wide variety of molecular types, including DNA, metabolites, mRNA, and cell surface molecules (1), which exert distinct influences on tumor development. Cell surface molecules play crucial parts in tumor proliferation and metastasis through adhesion mechanisms (2). Nectin-4 is a type I transmembrane adhesion molecule belonging to the immunoglobulin superfamily (3) and forms part of the nectin protein family together with Nectin-1, Nectin-2, and Nectin-3. Nectin-mediated cell adhesion is characterized by its independence from Ca^2+^ and its ability to engage in both homophilic and heterophilic interactions through its outer domain via *trans*-interactions (4–6). Nectin-1 and Nectin-2 are typically found in immune organs such as bone marrow, thymus, and spleen, whereas Nectin-3 is expressed mainly in the placenta and spermatozoa (4, 5). Under normal physiological conditions, Nectin-4 is expressed in the placenta and embryo, with low levels in healthy tissues and cells of adults (4). However, it has been reported to be highly expressed in various malignant solid tumors and to be associated with cancer progression and prognosis, establishing it as a novel cancer biomarker (7–10). Nectin-4 is involved in tumor cell development, including adhesion, proliferation, migration, and angiogenesis (7–11), and it has prognostic significance in numerous cancers (12–16). In 2019, the Food and Drug Administration (FDA) approved the first antibody-drug conjugate (ADC) targeting Nectin-4, known as enfortumab vedotin (EV), for treating urothelial carcinoma. Its clinical efficacy is a current research hotspot, as are its susceptibility to drug resistance and the potential adverse reactions that it can cause (17–19). In addition, its value in the diagnosis and prognosis of cancer is being widely explored. This review aims to compile and summarize the current state of research on Nectin-4, focusing on its diagnostic utility, prognostic significance, and its role in cancer treatment. Furthermore, the review offers an analysis of potential future research directions and prospective clinical applications for Nectin-4.

## 2 Overview of Nectin-4

**Figure 1:**
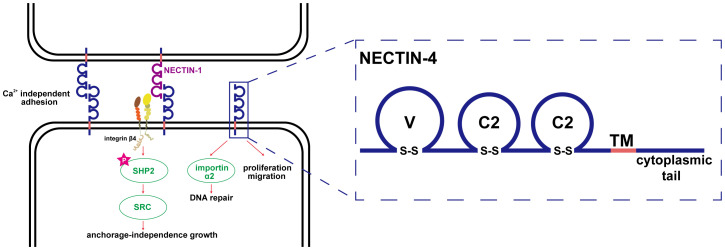
Biological function and structure of Nectin-4. The adhesion of nectins is Ca^2+^ independent, which is a homophilic and heterophilic process mediated by the outer domain through trans-interaction; the anchorage independence growth between tumor cells is achieved by PVRL4 through driving intercellular adhesion and stromal independent integrin-β4/SHP-2/c-Src activation, a process facilitated by the extracellular segment of Nectin-4; Nectin-4 can physically interact with importin-α2 to transfer to the nucleus to enhance DNA repair; the structure of Nectin-4 was shown in the right part of the figure.

Nectin-4 is a type I transmembrane polypeptide and a member of the immunoglobulin superfamily; it is encoded by the poliovirus receptor related-4 (PVRL4) gene and is 510 amino acids in full length (3, 20). The structure of Nectin-4, similar to other members of the nectin family, comprises an extracellular region, a transmembrane region, and a cytoplasmic tail, with the extracellular domain possessing three immunoglobulin-like loops (1 V-type and 2 C2-types) (21, 22). Notably, the adhesion of nectins does not depend on Ca^2+^, as it involves a homophilic and heterophilic process mediated by the outer domain through a *trans*-interaction (5). Notably, in addition to the traditional membrane protein form, Nectin-4 has a soluble form. Soluble Nectin-4 (43 kDa) is formed by shedding of the entire Nectin-4 extracellular domain under conditions of cellular hypoxia, a process in which a disintegrin and metalloproteinase (ADAM) has a vital role (23). Soluble Nectin-4 has been shown to interact with integrin-β4 on endothelial cells, promoting angiogenesis through signaling pathways involving Src, PI3K, Akt, and inducible nitric oxide (NO) synthase (24, 25). Initially, Nectin-4 was recognized as a viral entry receptor (22, 26–28). However, subsequent research unveiled its role as a cell adhesion protein closely related to the development of various diseases, in particular, cancers. The anchorage independence between tumor cells is achieved by PVRL4 through driving of intercellular adhesion and stromal independent integrin-β4/Src homology-2-containing protein tyrosine phosphatase 2 (SHP-2)/cellular Src activation, a process facilitated by the extracellular segment of Nectin-4 (7). In general, Nectin-4 shows little expression in healthy adult tissues, but it is highly enriched in embryonic and placental tissues. Nectin-4 is closely related to ectodermal development. Double allelic mutations in the PVRL gene cause ectodermal dysplasia-syndactyly syndrome 1, which is characterized by syndactyly (29–31). Several recent studies have confirmed that Nectin-4 is highly expressed in various solid tumors and is involved in their initiation and progression (32). Challita-Eid et al. used immunohistochemistry to analyze 2394 specimens from patients with various tumors, including bladder, breast, and ovarian cancers, and found that 69% of all samples stained positive for Nectin-4 (33). Although the precise mechanism by which Nectin-4 participates in cancer progression is unclear, some studies have shown that its intracellular region can physically interact with importin-α2 and transfer to the nucleus to enhance DNA repair, whereas the extracellular region can increase angiogenesis through various molecular pathways (34). In addition, several studies have confirmed the involvement of Nectin-4 in migration, adhesion, and proliferation of tumor cells (11, 35, 36). Nectin-4 is also involved in various benign diseases including endometriosis (37, 38). **Figure 1** shows the biological functions and structure of Nectin-4.

## 3 Molecular role of Nectin-4 in carcinogenesis

**Figure 2:**
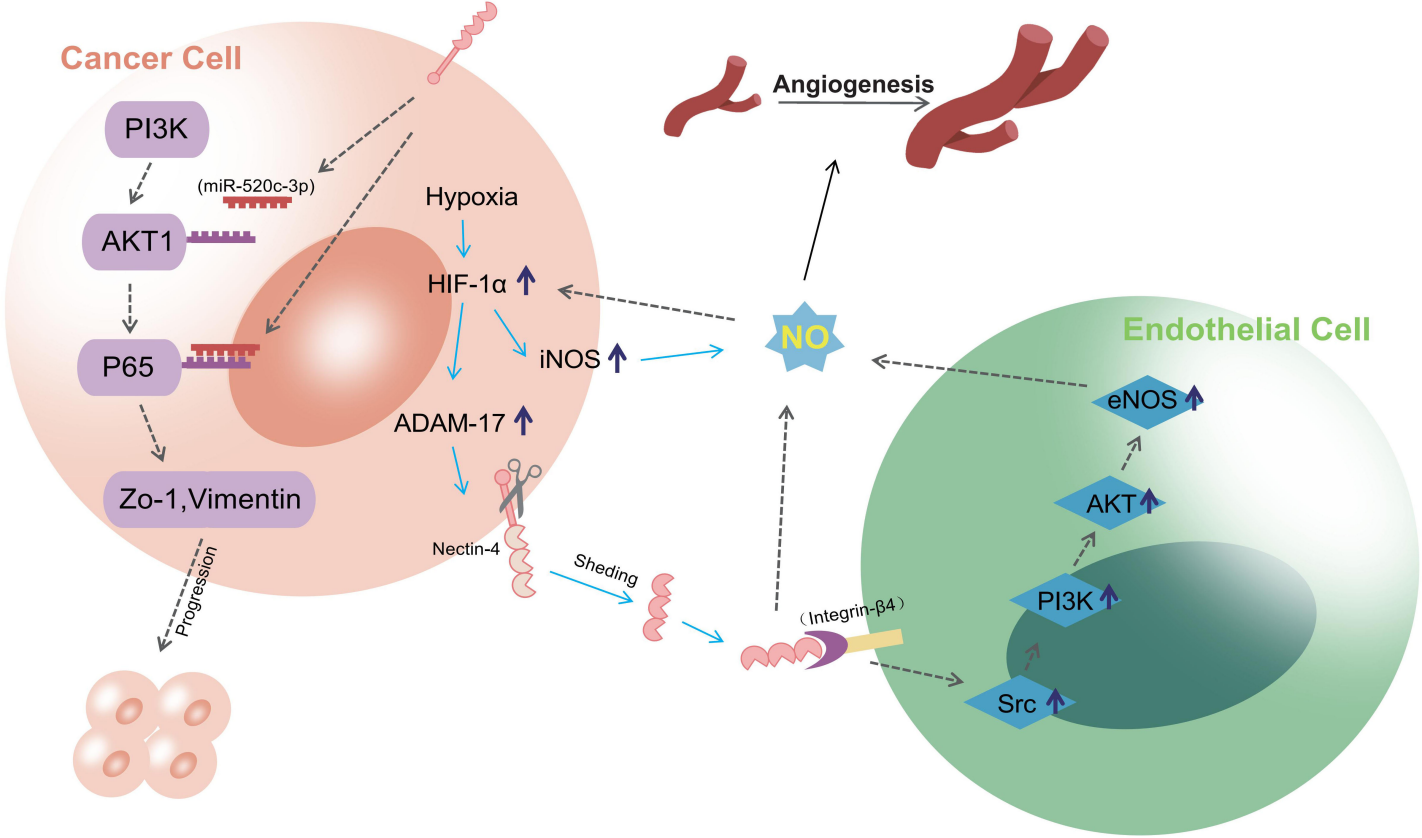
the process of Nectin-4 regulating cancer development through PI3K/AKT pathway and promoting angiogenesis after hypoxia. Nectin-4 can downregulate miR-520c-3p to activate the PI3K/AKT/NF-κB pathway, promoting the progression and metastasis; Soluble nectin-4, which is formed by the shedding of Nectin-4 extracellular domain during hypoxia, can interact with integrin-β4 on endothelial cells to promote angiogenesis. This progress is realized via the Src, PI3K, Akt, and eNOS pathways, and may be related to PI3K-Akt-mediated NO formation.

PI3K/AKT is a crucial signaling pathway involved in tumor development, regulating processes including cell invasion, proliferation, and apoptosis (39–41). Nectin-4 participates in several important processes in tumors through this pathway. For example, soluble Nectin-4, formed by the shedding of the extracellular domain under hypoxic conditions, interacts with integrin-β4 on endothelial cells to promote angiogenesis; this is achieved through the Src, PI3K, Akt, and endothelial NO synthase (eNOS) pathways (24) and may be related to PI3K-Akt-mediated NO formation. This was confirmed by a further study on oral cancer, in which addition of NO enhanced Nectin-4-mediated expression of eNOS and induced angiogenesis. However, Nectin-4 no longer induced angiogenesis after PI3K-Akt-mediated inhibition of the eNOS pathway (25). In addition, Nectin-4 has been reported to promote progression and metastasis of many tumors through the PI3K/AKT pathway. In breast cancer, Nectin-4 and tyrosine kinase receptor ErbB2 (also known as Her2) are highly expressed and can *cis*-interact with each other to activate the PI3K/AKT signaling pathway for DNA synthesis (42). Wnt/β-catenin signaling pathway is a self-renewal pathway in breast cancer stem cells. Siddharth et al. found that activation of this pathway was induced by soluble Nectin-4 through the PI3K/AKT axis and promoted the proliferation and metastasis of breast cancer cells (43). However, their study did not explore this process in detail. In August 2023, it was proposed that PI3Kα signaling may promote INPP4B-dependent lysosomal degradation, thereby enhancing activation of the Wnt/β-catenin signaling pathway (44). Moreover, Nectin-4 can downregulate miR-520c-3p, a microRNA, to activate the PI3K/AKT/nuclear factor kappa-B (NF-κB) pathway, promoting the progression and metastasis of osteosarcoma (45). In gastric and gallbladder cancers, Nectin-4 activates Ras-related C3 botulinum toxin substrate 1 (Rac1) through the PI3K/AKT pathway, promoting tumor proliferation and metastasis (46, 47). In addition to participating in tumor progression, Nectin-4 can mediate drug resistance through the PI3K/AKT pathway. Das et al. demonstrated upregulation of Nectin-4 in colon cancer cells after long-term treatment with standard chemotherapy agent 5-FU (48). Nectin-4 and AFADIN can form a complex that plays a part in intercellular junction formation (49). This complex can activate the PI3K/AKT cascade, leading to enhanced tumor cell survival and suppression of apoptosis (48). Thus, multiple studies have demonstrated that Nectin-4 is implicated in various aspects of tumorigenesis and progression via the PI3K/AKT pathway, suggesting potential novel strategies for future tumor treatment. **Figure 2** shows the process by which Nectin-4 regulates cancer development through the PI3K/AKT pathway and promotes angiogenesis after hypoxia.

Nectin-4 is involved in the transduction process of other signaling pathways in addition to the PI3K/AKT pathway. Normally, the extracellular domain of Nectin-4 also *cis*-interacts with the prolactin receptor, which is necessary for mammary follicle development, a process dependent on the Janus kinase 2-signal transducer and activator of transcription 5a (JAK2–STAT5a) signaling pathway (50). The JAK2–STAT5a pathway is vital for regulation of the growth of certain malignant tumors, including breast and prostate cancers (51). Nectin-4 also enhances p95-ErbB2-induced activation of the JAK–STAT3 signaling pathway (42) and cooperates with p95-ErbB2 to regulate expression of the sex determining region Y-box 2 (SOX2) gene, which in turn regulates the proliferation, survival, and differentiation of cancer cells, thereby enhancing the proliferation of breast cancer T47D cells (52). Moreover, Nectin-4 significantly promotes tumor-induced lymphangiogenesis and lymphatic metastasis by regulating the C-X-C motif chemokine receptor 4 (CXCR4)/C-X-C motif chemokine ligand 12 (CXCL12)–lymphatic vessel endothelial receptor-1 (LYVE-1) axis (53).These findings highlight the multifaceted involvement of Nectin-4 in various molecular pathways that contribute to the development and progression of cancer, underscoring its significance as a potential target for cancer therapy and research.

## 4 Diagnostic potential of Nectin-4

### 4.1 Serodiagnosis

Soluble and transmembrane forms of nectin proteins have been described in humans and rodents (4, 54–57). Soluble Nectin-4 was detected in the serum of patients with metastatic breast cancer and in the supernatants of breast cancer cell lines, with similar immunological and biochemical profiles, in a study demonstrating that the overexpression or silencing of tumor necrosis factor-α convert enzyme (TACE; also known as ADAM-17) enhances or reduces Nectin-4 shedding, respectively. Overexpression of TACE in breast cancer indicates that the formation of soluble Nectin-4, both *in vitro* and *in vivo*, can be attributed to TACE (58). Similarly, soluble Nectin-4 has been detected in the serum of patients with ovarian cancer, and it can be used, with CA-125, to identify benign ovarian disease and ovarian cancer (9). There is evidence that serum Nectin-4 levels are associated with the grade and progression of ovarian cancer (59). Notably, Nectin-4 can be detected in the early stages of ovarian cancer, even when CA-125 levels are not significantly elevated in the serum (60). Although soluble Nectin-4 has great potential and value in the diagnosis and monitoring of ovarian cancer, it still requires large-scale prospective studies for further validation and standardization.

Serum Nectin-4 may have diagnostic value for non-small-cell lung cancer (NSCLC). Takano et al. used mouse monoclonal antibodies (mAbs) to establish an enzyme-linked immunosorbent assay system to detect serum Nectin-4 levels. They found that serum levels of Nectin-4 in 164 patients with NSCLC were considerably higher than those of healthy volunteers; the percentage of Nectin-4 positivity in patients with NSCLC was 53.7%, whereas only three of the 131 healthy volunteers displayed false positivity, proving the superiority of Nectin-4 in terms of diagnostic sensitivity and specificity compared with carcinoembryonic antigen (CEA) and cytokeratin 19-fragment (CYFRA21-1) (8). However, it is worth noting that Nectin-2 seems to have an advantage over Nectin-4 in diagnosis of lung cancer (61). Thus, serum Nectin-4 is meaningful for NSCLC, but the evidence is not sufficient to support it as a serological marker.

As well as these potential applications in cancer diagnosis, increased levels of soluble Nectin-4 in serum may contribute to the diagnosis and monitoring of asthma (62) and may also represent a new biomarker for opioid dependence (63, 64). To date, most research has focused on Nectin-4 as a transmembrane protein of tumor cells, with less attention paid to soluble Nectin-4. More research is required to explore the serological value of soluble Nectin-4 in other cancers.

### 4.2 Target sites for molecular probes

Dean et al. designed an immuno-positron emission tomography (immunoPET) molecular probe targeting Nectin-4 for preclinical evaluation in tumor-bearing mice, based on the finding that Nectin-4 is highly expressed in several cancers with little expression in normal adult tissue (65). The probe was labeled with [89Zr] for AGS-22M6 (an anti-Nectin-4 mAb) and used for *in vivo* tumor detection in mice and [18F] for assessing the biodistribution of the probe in crab-eating monkeys (65). This was the first experiment in which a molecular probe was developed using Nectin-4 as a target. The new immunoPET probe developed in this study showed good detection of liver and bone lesions and could be used to evaluate expression levels of Nectin-4. In 2022, diagnostic and therapeutic pairs were developed based on an anti-Nectin-4 mAb, namely (99m)Tc-HYNIC-mAb (Nectin-4) and mAb (Nectin-4)-ICG. Of these, (99m)Tc-HYNIC-mAb (Nectin-4) has been used in immunological single-photon emission computed tomography for diagnosis and classification of triple-negative breast cancer (TNBC) and verified to have high targeting properties (66). mAb (Nectin-4)-ICG has been used for photothermal therapy of tumors. These imaging probes all use a mAb as the carrier; this shows good affinity but has a large molecular weight and slow blood clearance rate. To avoid the accumulation of imaging agents in organs, the selection of immune probe vectors may need to focus more on small-molecular-weight proteins such as antibody fragments or nanobodies in the future. A bicyclic peptide-based Nectin-4-targeted PET radiotracer, 68Ga-N188, was recently designed. Preclinical evaluation of this probe was completed in a mouse tumor model, followed by successful clinical translation to patients with advanced urothelial cancer and healthy volunteers (67). This marked the first clinical application of Nectin-4 as an imaging target. Although the value of Nectin-4 as a diagnostic target has been confirmed in clinical practice, the uptake of ^68^Ga-N188 in positive tumor lesions was moderate owing to its low affinity. The structure of N-188 needs to be further modified to improve its affinity with Nectin-4. Non-invasive monitoring of Nectin-4 expression could be beneficial for evaluating tumor progression and guiding treatment. This could benefit cancer patients with high expression of Nectin-4, such as those with locally advanced or metastatic urothelial carcinoma (la/mUC). It is expected that Nectin-4 will continue to be a focus of attention in the field of medical imaging, offering promising opportunities for improved diagnosis and monitoring of various diseases, particularly cancer.

## 5 Association between Nectin-4 and prognosis

Numerous studies have suggested a possible association between Nectin-4 expression and the prognosis of patients with various cancers, particularly breast cancer. Survivin is expressed in various tumor cells, and in breast cancer cells, it is correlated with the expression of Nectin-4. Both proteins are involved in tumor cell proliferation and differentiation, and in inhibition of tumor cell apoptosis, and they are both independent prognostic markers for breast cancer (68, 69). Therefore, exploring the interaction between Nectin-4 and survivin in breast cancer may provide value for the treatment of breast cancer in the future. A study on 197 patients with primary unilateral breast cancer (without involved lymph nodes or distant metastasis) found that 34 patients had Nectin-4 expression on the tumor cell membrane. Of the remaining 163 tumors that were negative for membrane Nectin-4 expression, 74.8% showed high cytoplasmic expression (12). This study also found that Nectin-4 positivity on the membrane and in the cytoplasm was associated with various survival indicators, indicating poor outcomes (12). TNBC tumor, which lack estrogen receptor, human epidermal growth factor receptor-2, and progesterone receptor, are more challenging to treat compared with other subtypes of breast cancer. Therefore, identifying new targets is crucial for assessing the prognosis of TNBC patients and guiding their treatment. Protein expression of Nectin-4 appears to be present in around 62% of TNBC cases and shows a strong correlation with mRNA expression levels. Moreover, high PVRL4 mRNA expression has independent negative prognostic value for metastasis-free survival in TNBC patients (13). Zeindler et al. used immunohistochemistry to explore the relationship between Nectin-4 expression and prognosis of TNBC patients. They found that high expression of Nectin-4 protein was correlated with lower tumor stage, better overall survival (OS), and negative lymph nodes and predicted better outcomes in patients with TNBC (70). Owing to the complexity of tumor cells, contradictory results may be obtained when using mRNA and protein expression levels of Nectin-4 to predict prognosis. More studies are needed to clarify the relationship between Nectin-4 and prognosis in TNBC.

Nectin-4 is associated with poor outcomes in various other cancers. For example, in pancreatic cancer, Nectin-4 shows significant correlation with the Ki67 value-added index and vascular endothelial growth factor expression, indicating its association with a prognosis (11). Deng et al. reported that increasing Nectin-4 expression in esophageal cancer cell lines promoted cell viability, migration, invasion, and tumor formation (14). This finding also supported the results of another study in which, among 94 patients with esophageal cancer, patients with increased Nectin-4 expression had shorter OS than those with low expression (71). Nectin-4 is upregulated in colorectal cancer tissues and associated with integrin β-1 expression and vasogenic mimicry formation (36). Vasogenic mimicry serves as an alternative method of arterial supply in tumors and is often associated with a poor prognosis. Strong expression of Nectin-4 is associated with high disease control rate (DCR) (72), and high expression of Nectin-4 in other cancers, including NSCLC, hepatocellular carcinoma, and gastric cancer, has been associated with poor patient outcomes (8, 15, 16). However, not all findings point in the same direction. For example, a study on luminal B breast cancer demonstrated a negative correlation between expression levels of Nectin-4 and survival time, but there was no statistically significant correlation with tumor differentiation, lymph node metastasis, histological subtype, or Ki-67 proliferation index (73). Moreover, Nectin-4 may not be suitable as a prognostic indicator in papillary renal cell carcinoma, in which it appears to have no statistically significant relationship with age, grade, or TNM stage (74). Expression level of a given protein may vary greatly between different tumors and can be affected by many factors including the diversity of tumor cells and the microenvironment. This could be why Nectin-4 does not show prognostic value in all tumor types. **Table 1** shows the associations of Nectin-4 with prognosis in different cancers.

**Table 1:** Association between Nectin-4 and tumours prognosis.

Cancer type	Number of patients	Nectin-4+%	prognosis	Year(Ref.)
Gallbladder cancer	68	63.2% +	reduced OS	2016/ (46)
Invasive breast cancer	140	64.3% +	poor prognosis	2011/ (68)
Luminal-A breast cancer	197	1.membrane Nectin-4: 17.3% + 2.cytoplasmic Nectin-4: 62% +	1.poor prognosis, reduced DFS and DRFS 2.prolonged DFS and LRFS	2014/ (12)
TNBC	290		poor prognosis, reduced MFS	2017/ (13)
TNBC	148	58% +	better OS	2019/ (70)
Luminal-B breast cancer(HER2 negative)	147		reduced OS, DFS and DRFS	2017/ (73)
Esophageal cancer	82	74.4% +	poor prognosis, reduced OS	2009/ (14)
Esophageal cancer	82	70% +	reduced OS	2019/ (71)
Urothelial cancer	47	59.6% +	higher DCR	2022/ (72)
Hepatocellular carcinoma	87	Cytoplasmic Nectin-4 67.82% +	reduced DFS and OS	2016/ (8, 15, 16)
Gastric cancer	212	membrane/cytoplasmic Nectin-4 60.4% +	reduced OS	2018/ (8, 15, 16)
Papillary renal cell carcinoma	190(type 1 of pRCC)	48.4% +	prolonged 5 years OS	2022/ (74)
	107(type 2 of pRCC)	36.4% +	independence	

TNBC, Triple-negative breast cancers; OS, overall survival; DFS, disease-free survive; DRFS, distance recurrence-free survive; LRFS, local recurrence-free survive; MFS, metastasis-free survival; DCR, disease control rate; pRCC, Papillary renal cell carcinoma.

## 6 Oncological therapeutic value of Nectin-4

### 6.1 Oncolytic measles virus

Oncolytic viruses, which are attenuated strains that can infect the body without causing serious disease, can utilize the immune system to recognize and kill tumor cells (75). Nectin-4 has long been a focal point for researchers as a specific therapeutic target, and its endocytotic characteristics have served as a foundation for various therapeutic studies (76). Measles virus (MV) has been a particularly active area of research in viral oncolytic therapy. An MV attenuated vaccine is an ideal candidate for oncolytic virus therapy and infects host cells by binding to three receptors: CD46, signaling lymphocytic activation molecule (SLAM), and Nectin-4. Fujiyuki et al. produced a recombinant MV (rMV-SLAMblind) that bound to Nectin-4 but not its original primary receptor SLAM; it also infected and suppressed the growth of subcutaneous xenograft models of various cancers, including breast cancers such as TNBC (77–80). Currently, seven early-stage clinical trials are actively exploring the effects of transgenic and vaccine strains of MV on head and neck cancer, multiple myeloma, and ovarian cancer (81). CD46 and Nectin-4 have been reported to be highly expressed on the surface of ovarian cancer cells, and MV has displayed a remarkable antitumor effect in a mouse model of ovarian cancer. Evanthia et al. used an oncolytic MV encoding the sodium iodide symporter (MV-NIS) gene to treat 16 patients with paclitaxel- and platinum-resistant ovarian cancer (82). The patients’ median OS (mOS) reached 26.5 months, and no dose-related adverse reactions were reported during high-dose treatment by injection. Immunological tests suggested that MV-NIS stimulated the body’s cellular immunity to mediate the antitumor effect (82). Considering the limited clinical success rate, scholars are exploring methods to strengthen MV-induced viral oncolytic therapy. For example, studies have demonstrated that small molecules such as baicalein and cinnamaldehyde can enhance the oncolytic effects of MV strains on breast cancer (83). Furthermore, ursolic acid (UA) is a phytochemical with great potential in the treatment of breast cancer, and UA nanoparticles developed by nanoemulsification technology have exhibited enhanced drug solubility. A combination of UA and MV therapy was reported to enhance the killing of breast cancer cells by increasing autophagic flux, thereby providing an improved therapeutic effect (84). Although oncolytic viruses have the potential to treat cancer, the complexity and heterogeneity of cancer cells make it impossible to completely kill them by this treatment alone. Developing personalized treatment plans based on patients’ immune status and accurately delivering drugs to the tumor site are the most urgent aspects to be addressed to improve therapeutic effects.

### 6.2 EV-ADC targeting Nectin-4

**Figure 3:**
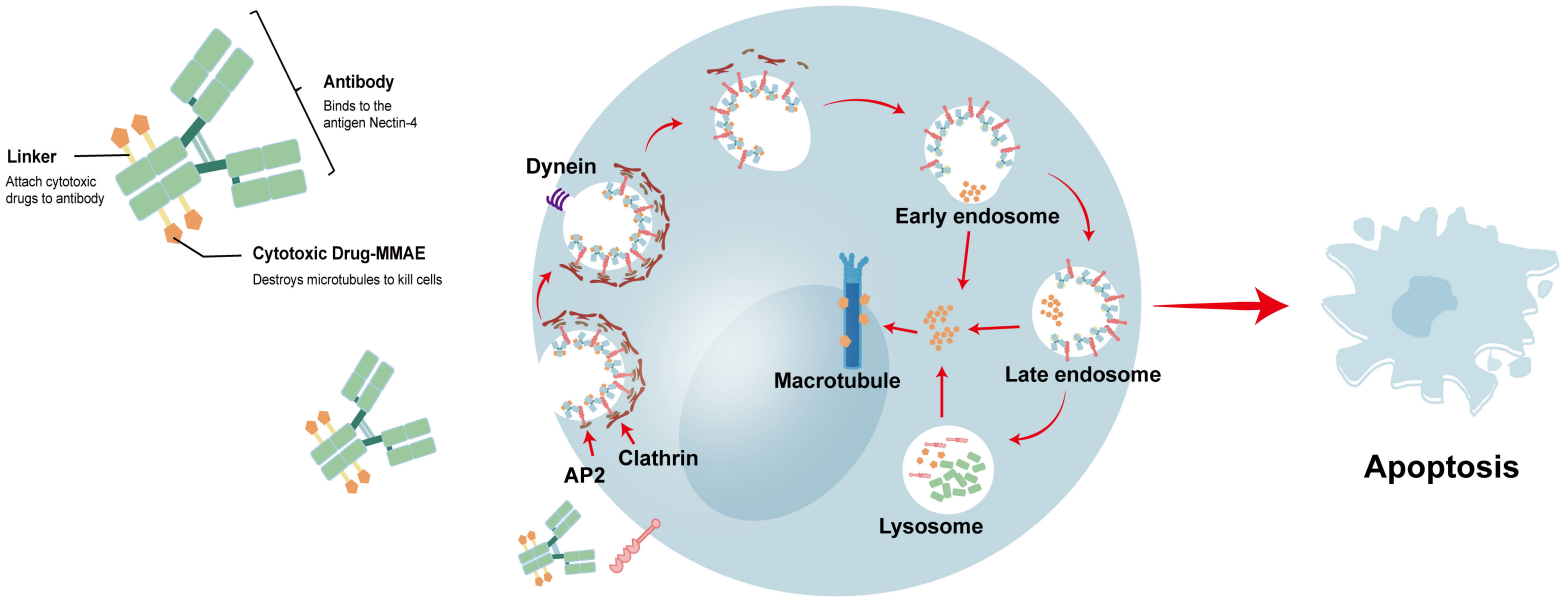
the structure of EV and the process of destroying cancer cells. EV binds to Nectin-4 on the cell membrane, and the EV-antigen complex is internalized and transported along the endosomal pathway. In this process, various enzymes can incompletely hydrolyze EV, and finally EV is completely decomposed by lysosomes. The released MMAE will destabilize the microtubules, and the cells will undergo apoptosis due to the inhibition of division.

ADCs combine a small-molecule anticancer agent with a mAb to selectively deliver cytotoxic agents to tumor targets with increased efficacy and reduced toxicity. Nectin-4 has emerged as an attractive target for ADC therapy, and in 2019, the FDA approved the first ADC (EV) targeting Nectin-4 for the treatment of urothelial carcinoma (18). EV is a complex formed by the protease-dependent linker binding microtubule-disrupting agent monomethyl auristatin E (MMAE) with anti-Nectin-4 mAb (AGS-22M6) (85). EV binds to Nectin-4 on the cell membrane, and the EV–antigen complex is internalized and transported along the endosomal pathway. During this process, EV is initially hydrolyzed by various enzymes, before finally being completely decomposed by lysosomes. This releases MMAE, which destroys microtubules, causing the cells to undergo apoptosis owing to inhibition of division (18, 76, 86). **Figure 3** shows the structure of EV and the process by which it destroys cancer cells. In preclinical research, EV substantially inhibited tumor growth in a mouse xenograft model of human bladder, breast, lung, and pancreatic cancers (33). This outcome has driven the translation of EV into clinical research.

### 6.3 Clinical trials of EV

EV-101 (NCT02091999) was a trial conducted in patients with mUC, with the primary objectives of evaluating drug safety, pharmacokinetics, and patient tolerance. A total of 155 patients were recruited for the EV-101 study. In phase I, patients received incremental doses of EV of up to 1.25 mg/kg on days 1, 8, and 15 of each 28-day cycle (87). Encouraging survival data from this phase suggest a recommended phase 2 dose of 1.25 mg/kg on days 1, 8, and 15 of every 28 days. Further, 112 patients received EV at the preliminary recommended phase 2 dose. In this study, the objective response rate (ORR) was 43%, the mOS was 12.3 months, and the median progression-free survival (mPFS) was 5.4 months. The most common treatment-related adverse events (TRAEs) were rash, peripheral neuropathy, and fatigue. Four deaths occurred owing to continued treatment in this study (respiratory failure, urinary tract obstruction, diabetic ketoacidosis, and multiorgan failure) (87). Overall, the EV-101 study provided valuable insights into the potential of EV as a treatment option for mUC, and its safety and efficacy data were promising for further clinical development. In another smaller trial (NCT03070990), 17 Japanese patients with refractory urothelial cancer were randomly assigned to receive either 1 or 1.25 mg/kg of EV for a standard cycle of 28 days. The adverse reactions seen in this study were similar to those reported in the EV-101 phase I trial, with an mPFS of 8.1 months (88).

EV-201 (NCT03219333) study was a global, phase II study designed to evaluate the drug’s treatment effect and was key to the FDA’s decision to approve EV for treatment of patients with advanced urothelial cancer. This study was a single-arm trial with two separate patient cohorts. Cohort 1 included 125 patients treated with EV at a standard dose; the ORR was 44%, including 12% complete response (89, 90). Cohort 2 included 89 patients (91), who were treated with EV at standard doses and had an ORR of 52% and a complete response rate of 20%. TRAEs were acute kidney injury, hyperglycemia, decreased appetite, hypotension, diarrhea, neutropenia, and skin reactions. Four patients died of treatment-related toxicity (acute kidney injury, metabolic acidosis, multiorgan failure, and pneumonitis) (91). The EV-201 study played a vital part in demonstrating the efficacy and safety of EV for patients with advanced urothelial carcinoma. The study showed promising clinical outcomes, including significant ORRs and complete responses in both cohorts.

The EV-301 (NCT03474107) trial was a randomized phase III clinical trial designed to evaluate OS in la/mUC patients after treatment with EV. This trial aimed to assess the efficacy of EV compared with standard chemotherapy. It enrolled 608 patients, 301 of whom were treated with EV at standard dose, whereas the other 307 received chemotherapeutic agents (standard doxorubicin, paclitaxel, or vinblastine) on day 1 of a 21-day cycle. The mOS was 12.88 months in the EV group and 8.97 months in the chemotherapy group. The confirmed ORR in the EV group was 40.6%, which was consistent with results of previous studies and substantially higher than the ORR of 17.9% for the chemotherapy group. However, the incidence of adverse reactions in the two groups was similar, indicating that the two treatment modalities had a comparable profile in terms of safety. The study reported a low rate of complete responses (92), although EV was highly effective in reducing tumor size and extending OS. The EV-301 trial provided compelling evidence that EV significantly extends OS in patients with la/mUC.

A combination of EV with pembrolizumab (an immune checkpoint inhibitor) demonstrated enhanced treatment outcomes compared with EV alone (90, 93). EV-103 (NCT03288545) evaluated the efficacy, tolerability, and safety of this combination. The dose escalation phase of the study established the safe dose of EV to be 1.25 mg/kg. Subsequently, 40 patients with la/mUC received EV + pembrolizumab. This trial reported the highest mOS (26.1 months) in a first-line urothelial carcinoma trial (90, 93, 94). EV-103 cohort K enrolled 149 patients who were randomly assigned to receive either EV in combination with pembrolizumab or EV monotherapy (95). After 3 months of follow-up, the ORR of the EV + pembrolizumab treatment group was 64.5%, and no new safety issues occurred (96). The results of EV-103 confirmed the advantages of immunotherapy combined with ADC therapy compared with ADC monotherapy. EV-302 (NCT04223856) was an open, global, randomized controlled study. In this clinical trial, 886 treatment-naive patients with la/mUC who met the eligibility criteria for cisplatin or carboplatin were enrolled. They were randomized to receive either EV + pembrolizumab or chemotherapy. The mOS and median progression-free survival of the EV + pembrolizumab group were twice those of the chemotherapy group (97). Thus, the results of EV-302 have changed the standard of care for bladder cancer (98), challenging conventional first-line treatments (including platinum-based chemotherapy) for patients with la/mUC and further validating the efficacy of combined immunotherapy and targeted therapy.

The FDA has progressively approved new indications for EV as clinical trials have been conducted. In 2019, based on the EV-201 cohort 1 study, EV was approved for the treatment of adult patients with la/mUC who had received a PD-1 or PD-L1 inhibitor and platinum-containing chemotherapy. After clinical trials of the EV-201 cohort 2 and EV-301 had been completed, the FDA extended the indications of EV to patients ineligible for cisplatin-containing chemotherapy who had previously received one or more prior lines of therapy. EV-302 showed that EV + pembrolizumab was superior to first-line chemotherapy, which led the FDA to approve EV combined with pembrolizumab for patients with la/mUC. FDA had previously granted accelerated approval to this combination for patients with la/mUC who were ineligible for cisplatin-containing chemotherapy. These consecutive approvals of EV demonstrate its established position in the treatment landscape of la/mUC.

Some new clinical studies are being conducted to broaden the indications of EV. Sacituzumab govitecan (SG), which targets TROP2, and EV are both FDA-approved ADCs. TROP2 and Nectin-4 are highly expressed in mUC. Clinical trial NCT04724018 was the first study to combine two ADCs for the treatment of mUC. This study recruited 24 mUC patients with disease progression after chemotherapy or immunotherapy. Different combinations of SG and EV doses were designed, and the recommended doses (SG: 8 mg/kg, EV: 1.25 mg/kg) for the phase II study were determined. The ORR in this study was 70%, and 78% of patients had adverse events of grade ≥3 (99). Overall, although the SG + EV combination therapy has shown promising clinical significance, the incidence of adverse reactions was high. EV + SG combination therapy may therefore not be suitable for further development. Clinical trials (EV-303; NCT03924895) of EV combined with pembrolizumab as a perioperative neoadjuvant therapy in patients with muscle-invasive bladder cancer are also underway (100) and may provide new options for patients who are not candidates for cisplatin therapy during the perioperative period.

The therapeutic efficacy of EV in patients with urothelial carcinoma has shown variations based on the histological and molecular characteristics of the tumors, particularly expression levels of Nectin-4. In the real-world UNITE dataset, the ORR was 58% in patients with pure urethral histology and 42% in patients with any variant component (101). The expression of Nectin-4 in urothelial carcinoma tumors can vary significantly. Jean et al. detected the expression of Nectin-4 in 169 patients with urothelial carcinoma by immunohistochemistry. Overall, 87% of non-muscle-infiltrating urothelial cancer samples and 58% of muscle-infiltrating tumors were positive. This variation suggests that Nectin-4 expression levels may influence the efficacy of ADC treatment. Nectin-4 expression levels have been associated with different subtypes of urothelial carcinoma. A study using whole-transcriptome RNA sequencing found that most sarcomatoid carcinomas and all but two small-cell carcinomas expressed low levels of Nectin-4 mRNA (102). Nectin-4 expression varies considerably between subtypes, and its expression is correlates with ADC treatment efficacy (103). Considering the differences in tissue expression, testing for Nectin-4 expression levels prior to ADC treatment needs to be considered to optimize treatment efficacy. In the future, EV may be used for the treatment of different tumor subtypes with treatment tailored according to Nectin-4 expression.

Further studies have confirmed that the potential applications of EV are not limited to urothelial carcinoma. Rabet et al. selected one of six mAbs with the best affinity to develop an ADC for Nectin-4, which was used for targeted therapy in TNBC xenograft mouse models. A remarkable therapeutic effect was achieved, which was related to the dose and expression level of Nectin-4 (13). Clinical trials of EV for pancreatic cancer, prostate cancer, and squamous cell carcinoma of the penis are also underway (NCT05915351, NCT04754191, NCT06104618). In addition, EV has shown favorable results in the treatment of skin cancer (104). We anticipate that EV will deliver good efficacy and safety for a broader range of cancers in the future. **Table 2** displays the details and results of EV clinical trials.

**Table 2:** Clinical trial of EV for the treatment of patients with UC.

Cinical trials	Number of patients	Characteristic of patients with UC	Purpose	Treatment	Result	Year(Ref.)
EV-101 phaseI (NCT02091999) EV-101 phaseII (NCT02091999)	155	received chemotherapy or ICI disease progressed	Safety and tolerability	EV, 0.5 -1.25 mg/kg (Schedule A)	ORR: 43%, duration of response: 7.4 months, mOS: 12.3 months	2020/ (87)
	112			EV, 1.25 mg/kg (Schedule A)		
Takahashi S et al. (NCT03070990)	17	refractory urothelial cancer patients		EV, 1 or 1.25 mg/kg (Schedule A)	in the whole population: ORR: 35.3%, DCR: 76.5%; in Arm A: ORR 44.4%, DCR 100%; in Arm B: ORR 25%, DCR 50%	2020/ (88)
EV-201 cohort 1 (NCT03219333)	125	received chemotherapy or ICI, and then disease progressed	efficacy and safety	EV, 1.25 mg/kg (Schedule A)	ORR: 44%, complete response: 12%	2019/ (89)
EV-201 cohort 2 (NCT03219333)	89	Contraindication to chemotherapy	efficacy and safety	EV, 1.25 mg/kg (Schedule A)	ORR: 52%, complete response: 20%	2021/ (91)
EV-301 (NCT03474107)	608	received chemotherapy and ICI, and then disease progressed	clinical benefit	EV group: EV, 1.25 mg/kg (Schedule A) Chemotherapy group: doxorubicin, paclitaxel, or vinblastine (Schedule B)	EV group: OS 12.88 months, mPFS 5.55 months, ORR 40.6%; Chemotherapy group: OS 8.97 months, mPFS 3.71 months, ORR 17.9%	2021/ (92)
EV-103 dose escalation/cohort A (NCT03288545)	45	Contraindication to chemotherapy	Safety and tolerability	Dose Escalation Phase (N = 5): EV, 1-1.25 mg/kg (Schedule C) + P, 200mg (Schedule B) Dose Expansion Cohort A (N = 40): EV: EV, 1.25 mg/kg (Schedule C) + P, 200mg (Schedule B)	DCR: 93.3%, ORR: 73.3%. At the first tumor assessment: mPFS 12.3 months, mOS 26.1 months	2023/ (93)
EV-103 cohort K (NCT03288545)	149	Contraindication to chemotherapy	Efficacy and safety	Group1(N=76): EV, 1.25 mg/kg (Schedule C) + P, 200mg (Schedule B) Group2(N=73): EV, 1.25 mg/kg (Schedule C)	Group1: ORR 64.5%, CR 10.5%, PR 53.9%, ORR 49%, mOS 22.3 months Group1: ORR 45.2%, CR 4.1%, PR 41.1%, ORR 33%, mOS 21.7 months	2023 (95, 105)
EV-302 (NCT04223856)	886	previously untreated and met the treatment requirements of cisplatin or carboplatin		Group1: EV, 1.25 mg/kg (Schedule C) + P, 200mg (Schedule B) Group2: gemcitabine with cisplatin or carboplatin	Group1: ORR 67.7%, mOS 31.5 mo, mPFS 12.5 Group2: ORR 44.4%, mOS 16.1 mo, mPFS 6.3	2023 (97)
McGregor BA et al. (NCT04724018)	24	patients with disease progression after chemotherapy or immunotherapy	Safety and tolerability	SG + EV, dose of each group is different (Schedule C)	ORR: 70%	2024 (99)

OS, overall survival; DCR, disease control rate; mOS, median OS; ICI, immune checkpoint inhibitor; ORR, objective response rate; mPFS, median progression-free survival; CR, complete response; PR, partial response; P, pembrolizumab.

Schedule A is days 1, 8 and 15 of a 28-day cycle. Schedule B is day 1 of a 21-day cycle. Schedule C is days 1 and 8 of a 21-day cycle.

### 6.4 EV-related TRAEs

Severe TRAEs are a key factor leading to dose reduction or discontinuation of EV treatment. The predominant adverse events (≥20%) of all grades include fatigue, peripheral neuropathy, decreased appetite, rash, alopecia, nausea, dysgeusia, diarrhea, dry eye, pruritus, and dry skin. Warnings for EV encompass hyperglycemia, peripheral neuropathy, ocular disorders, skin reactions, infusion site extravasations, and embryo-fetal toxicity. The most common AEs leading to discontinuation or dosage reduction are peripheral neuropathy and rash. The types of peripheral neuropathy are diverse and predominantly characterized by sensory abnormalities, which can be attributed to the microtubule toxicity of MMAE. There are many types of rashes, with maculopapular rashes being the most common and severe. Several clinical trials have shown that reducing the dosage and using steroids can have a therapeutic effect on rash. Grade ≥3 adverse events include maculopapular rash, febrile neutropenia, anemia, and hyperglycemia. Hyperglycemia is common in patients with a history of hyperglycemia or BMI ≥30 kg/m^2^; therefore, it is important to prevent this complication in overweight patients. Dry eye symptoms were observed in 36% of patients, and 14% of patients experienced blurred vision possibly related to dry eye symptoms. The U.S. Package Insert) recommends the use of artificial tears as a preventive measure for dry eyes or the use of ophthalmic corticosteroids after ophthalmic examinations. During follow-up periods, most patients’ TRAEs gradually resolved or improved. There have been a few cases of deaths associated with EV treatment. Each death event was complex and involved multiple confounding factors such as underlying conditions and previous treatments.

### 6.5 Drug resistance to EV

A majority of patients develop resistance to EV during treatment. Anti-cancer drug resistance encompasses intricate mechanisms that include reduced antigen expression, induction of drug transport proteins, and trafficking defects, as well as alterations in signaling and apoptotic pathways (106). Several studies have demonstrated that membrane Nectin-4 expression is decreased in metastatic tissues, and reduced or absent membrane Nectin-4 expression in mUC tissues may have a crucial role in EV resistance (107, 108). However, certain studies have confirmed the continued high expression of Nectin-4 in new tissue specimens after cancer recurrence (102), which does not support a decrease in antigen levels as key to the emergence of resistance to EV. Cabaud et al. investigated the causes of EV resistance using a mouse model of breast cancer (109); this is the only study of the mechanisms of EV resistance. They reported that ABCB1, which encodes the ATP-binding transporter multidrug resistance 1 (MDR-1)/P-glycoprotein (P-gp), was upregulated in EV-resistant tumors, and sensitivity to EV targeting Nectin-4 was restored in *in vitro* and *in vivo* models after P-gp was inhibited. In addition, the combined use of a third-generation P-gp inhibitor and ADC was well tolerated in mice and restored sensitivity to ADC (109). Considering the limitations of preclinical models, data from clinical patient populations are now urgently needed for further exploration and confirmation.

In summary, resistance to EV treatment is a complex phenomenon that may involve various mechanisms. The role of P-gp and its inhibition in overcoming EV resistance has shown promise in preclinical models (109). However, clinical data are essential to fully understand resistance mechanisms and develop effective strategies to combat resistance in patients receiving EV therapy.

### 6.6 Other ADCs targeting Nectin-4

Other ADCs targeting Nectin-4 include the novel 9MW2821, which consists of an anti-Nectin-4 antibody (MW282), a linker (IDconnect), and MMAE. IDconnect is a novel thioether linker that cross-links the Fab and hinge regions, resulting in a more robust attachment of MMAE (110). The design of IDconnect is the strength of 9MW2821, which shows reduced amounts of off-target drug in circulation and decreased liver toxicity. Compared with EV, 9MW2821 has a homogeneous drug–antibody ratio (DAR) and has shown better antitumor effects and fewer adverse effects in a preclinical trial (110). Research and development objectives for 9MW2821 are focused on mitigating the adverse reactions caused by EV and improving the therapeutic effect in patients with la/mUC. Clinical trials of 9MW2821 are about to begin (NCT06079112 and NCT06196736). Two clinical studies have expanded from la/mUC patients to patients with other malignant solid tumors (NCT05216965 and NCT05773937). ADRX-0706 and BAT8007 are also ADCs targeting Nectin-4. ADRX-0706 has a DAR of 8 (the DAR of ADCs is usually 2-4), leading to a greatly increased drug concentration in the local area of the tumor. Therefore, the therapeutic effects obtained with high DARs should receive more attention. A clinical trial of ADRX-0706 is being carried out in patients with specific advanced solid tumors, including urothelial carcinoma, head and neck squamous cell carcinoma, breast cancer, cervical cancer, ovarian cancer, NSCLC, and pancreatic cancer (NCT06036121). The cytotoxic drug carried by BAT8007 is a topoisomerase I inhibitor (Exatecan) that interferes with DNA replication and transcription to induce apoptosis. It differs from other ADCs targeting Nectin-4 by replacing MMAE, thereby changing the mechanism of tumor cell killing. At present, a clinical trial of BAT8007 is recruiting patients with advanced solid tumors (NCT05879627). The potential applications of these three new drugs targeting Nectin-4 have already been extended to various malignant solid tumors, underscoring the widespread attention paid to Nectin-4 and recognition of its potential as a therapeutic target. It is expected that research on Nectin-4 in the field of cancer therapy will continue to grow and progress.

### 6.7 BT8009 and BT7480

Not all ADCs translate perfectly from preclinical models to humans, in part because of poor drug penetration and long drug half-lives (days to weeks). Although antibodies provide high affinity and highly specific carriers for toxin delivery, their large molecular size (150 kDa) hinders their ability to overcome the combined barriers of the endothelial barrier, interstitial pressure, and complex stromal structures in tumors (111). This results in low extravasation rates and slow diffusion outside the cell. Only 0.01–0.10% of injected antibodies may reach solid tumor antigens (111). Bicyclic toxin conjugates (BTCs) targeting Nectin-4 offer an alternative to traditional ADCs by utilizing small hydrophilic peptides instead of mAbs. This allows BTCs to quickly diffuse from the systemic circulation and potentially overcome some of the limitations associated with ADCs. BT8009 is composed of a bicyclic peptide that binds to Nectin-4, a cleavable adaptor system, and a cell-penetrating toxin, MMAE. It has demonstrated superior or comparable antitumor activity compared with EV in several models. BT8009 has the advantage of fast clearance and can penetrate tumors and target tumor cells (112). In summary, BT8009 offers a promising alternative to traditional ADCs for targeting Nectin-4 and other cancer-associated molecules. Clinical trials aimed at further assessing its effectiveness and safety in real-world patient populations are in progress.

BT7480 is composed of two CD137 bicycles and a Nectin-4 bicycle. CD137 is an inducible costimulatory receptor that can participate in the activation of a variety of immune cells and has been reported to be a promising target for cancer immunotherapy (113, 114). Transcriptional analysis has shown that Nectin-4 and CD137 are co-expressed in a variety of cancers. BT7480 targets Nectin-4-expressing tumor cells and then binds to and agonizes CD137 on nearby immune cells to remodel the tumor immune microenvironment, thereby eliminating tumor cells and inhibiting tumor recurrence (115, 116). Compared with CD137 activation alone, immune killing after targeted activation shows a better therapeutic effect and less liver toxicity.

## 7 Conclusion

This review provides an overview of the role of Nectin-4 in cancers, focusing on clinical trials related to Nectin-4-targeted tumor treatments and the development of novel drugs targeting Nectin-4. Nectin-4 is upregulated in multiple tumor types and promotes tumorigenesis, development, and angiogenesis through pathways such as PI3K/AKT. Nectin-4 has shown clinical significance in serological and molecular imaging diagnosis of some tumor types, such as ovarian cancer, urothelial carcinoma, and breast cancer, but more studies with larger sample sizes are needed to promote its further applications. In 2019, EV was approved by the FDA as the first ADC drug targeting Nectin-4 for the treatment of la/mUC. Subsequently, various clinical trials related to EV have reported encouraging survival data, which has led to the continuing expansion of EV indications in la/mUC. These results have promoted the development of new drugs targeting Nectin-4 and have clearly demonstrated its value as a therapeutic target for tumors. However, TRAEs and drug resistance are aspects that require significant attention. Common TRAEs include rash, peripheral neuropathy, and fatigue, and close monitoring during initial treatment cycles is advisable. Moreover, there has been no definitive study to elucidate the exact underlying causes of drug resistance, which is a key factor impeding further treatment with EV. New therapeutic drugs targeting Nectin-4 have been improved in terms of linkers and DAR compared with EV; however, the relevant clinical trials are still underway, so the treatment efficacy of these alternatives remains unknown.

In conclusion, Nectin-4 is a highly valuable molecular target in cancer, deserving further research and development.

## Author contributions

KL: Data curation, Writing – original draft. YZ: Writing – original draft, Funding acquisition. MZ: Writing – review & editing, Visualization, Data curation. XJ: Writing – review & editing. XL: Writing – review & editing, Conceptualization.
